# The Atmosphere of the Central London Railway

**Published:** 1903-07-11

**Authors:** 


### THE ATMOSPHERE OF THE CENTRAL LONDON RAILWAY.

THE report issued some time ago by the London County Council on the atmosphere of the Central London Railway has something of an historical flavour about it, quite unintended by its authors, of whom one completed his work as long ago as last July and the other in early November. It reads much as might an "appreciation," studiously impartial in tone, of former conditions in South Africa by a political economist in Europe. The Tube authorities will seize upon any details that may suit them under present circumstances, and the former "outlander," otherwise the Tube's patron and victim, will smile grimly over an attempt by experts to describe a condition of things of which his own knowledge was intimate and personal, though unscientific.

The report relates to the atmosphere of the Tube as it was between the months of March and early November last year, and deals with it from three points of view, viz., the amount of carbonic acid present, the amount of organic matter, and the number and general characters of the micro-organisms it was found to contain. Eighty experiments were made by Dr. Clowes to determine the first two factors, while Dr. Andrewes, Bacteriologist of St. Bartholomew's Hospital, founded his report upon an analysis of twelve samples of as exhaustive a character as the general nature of the inquiry would permit.

Both observers have dealt separately with air taken at different hours of the day and from several localities, such as the lifts, the passages, the stations, the tunnels, and the carriages. This minute subdivision is doubtless interesting scientifically, and to those responsible for the ventilation of the Tube will certainly save much trouble. What, however, is alone interesting to other people, or of any special importance from the point of view of the public health, is the air that the traveller breathes in the carriages. Over this he has not, as in an ordinary carriage, any control whatever, and good or bad he has to breathe it for periods varying from a few minutes to half an hour, as often as he uses the Tube. In future work on the subject it would be much better if the Council directed its agents to confine themselves to this point alone; in which case more work would be possible and no side issues could be raised. Dr. Clowes' summary of his carbon dioxide results is that about 22 per cent. of the samples show "less than twice as much as that in the outside air, and 34 per cent. less than two and a half times as much."

From our point of view, however, the results are much worse than this, since taking the carriage and lift air alone we find the percentage of carbon dioxide is 0·116, or nearly 12 volumes per 10,000, *i.e.*, three times more than normal air. This cannot be considered anything but a very bad result, since on this railway there is nothing except exhalation from human bodies to account for the excess. Dr. Clowes has not attempted to summarise his "organic matter" results, and examining them for ourselves we note that they are so exceedingly irregular as to suggest once more that, as has frequently been stated before, the acid permanganate method of estimation is very unreliable, though readily applied. In future researches it would be much more satisfactory to take steps to return the organic matter present as ammonia. The deductions from the organic matter are of great importance because practically direct, while those from the carbon dioxide are merely inferential.

Turning now to the bacteriological analysis, we get results which, though not striking in themselves, represent in the present state of knowledge of atmospheric bacterial flora a very troublesome inquiry. The general average of organisms present is not very much against the Tube, but the difference is great if those of the mesophile class are alone considered. The presence of these in such quantity, though they appear to have been mainly sarcinæ, and therefore not pathogenic, is decidedly significant. Nor can any stress be laid upon the fact that bacteria known to be directly productive of disease were not found, since in the limited amount of air used in a general "botanical" inquiry of this sort they were not likely to be. Further it must not be forgotten that the breathing of foul air causes a general loss of tone independently of the production of particular disease. All these inquiries, however, relate to the past, since when many alterations in ventilation have been made. How far they are effective is not yet certain, but the general opinion is that there is some improvement. When the alterations are quite complete the Council's experiments might well be repeated. Too much importance, however, should not be attached to them, nor the public express itself satisfied, until it is no longer possible to recognise a Tube station simply by the smell and there is no stuffiness perceptible while travelling.

